# Delineation of Subarachnoid Cisterns Using CT Cisternography, CT Brain Positive and Negative Contrast, and a Three Dimensional MRI Sequence: A Pictorial Review

**DOI:** 10.7759/cureus.23741

**Published:** 2022-04-01

**Authors:** Santosh Rai, Saubhagya Srivastava, Mayur Kamath, B V Murlimanju, Geetanjali Parmar, Gowthami Chebrolu

**Affiliations:** 1 Department of Radiology, Kasturba Medical College, Mangalore, Manipal Academy of Higher Education, Manipal, IND; 2 Department of Neurosurgery, Kasturba Medical College, Mangalore, Manipal Academy of Higher Education, Manipal, IND; 3 Department of Anatomy, Kasturba Medical College, Mangalore, Manipal Academy of Higher Education, Manipal, IND

**Keywords:** anatomy, radiology, pneumocephalus, subarachnoid hemorrhage, 3d ciss mri, ct brain, ct cisternography, subarachnoid cisterns

## Abstract

The basic anatomy and morphology of subarachnoid cisterns of the brain are interesting and challenging topics with high clinical significance. These enlarged CSF-filled expansions are important as they transmit various neurovascular structures. The cisterns can be classified based on their location as supratentorial, at the level of the tentorium, and infratentorial. They are also classified as paired and unpaired cisterns. The anatomical and radiological information about the cisterns is clinically and surgically relevant in diagnosing and managing many neurological disorders. It is also essential in medical teaching. This pictorial essay reviews the radiological images where the subarachnoid cisterns are delineated in four unique circumstances.

## Introduction and background

The subarachnoid cisterns are clinically and surgically significant enlarged cerebrospinal fluid (CSF)-filled pockets of the subarachnoid space that transmit important neurovascular structures including cranial nerves and intracranial vessels. The pia and arachnoid mater are closely approximated over the convexities of the brain (e.g., the cortical gyri). However, although the concavities of the brain are closely approximated by the pia mater, the arachnoid mater only loosely approximates the concavities of the brain. Thus, the subarachnoid space varies in depth and the more expansive spaces form the subarachnoid cisterns [1]. Yasargil provided the first in-depth description of the cisterns in the 1970s, highlighting their limits, relations, and contents [2,3]. Cisternal anatomy of the brain is an interesting and challenging topic of high clinical significance. Radiological evaluation of the cisterns is limited in normal studies, however, in the presence of accumulations or pathologies, the radiological visualization of cisterns is enhanced [4].

In this study, we deeply reviewed the radiological images where the subarachnoid cisterns are delineated in four unique case scenarios: a case of suspected CSF rhinorrhea who underwent computed tomography (CT) cisternography, CT brain with negative contrast demonstrating a case of extensive pneumocephalus, CT brain with positive contrast demonstrating a case of subarachnoid hemorrhage (SAH), and a patient with trigeminal neuralgia who underwent routine three-dimensional (3D) constructive interference in steady state (CISS) MRI of the brain. 

CT cisternography was performed for a 35-year-old female who presented with watery nasal discharge to evaluate for suspected CSF rhinorrhea [5]. CT cisternography is a standard imaging modality that is used to diagnose the occult site of CSF leak in cases of CSF rhinorrhea [6]. A pre-contrast plain CT is first carried out to identify areas of defects in the cribriform plate, roofs of ethmoidal cells, or skull base. Three to 10 mL of iodinated nonionic contrast agent is then administered into the thecal sac, following which the patient is placed in Trendelenburg position for opacification of cisterns, and CT scan is performed with thin slices in coronal and axial planes in both prone and supine positions [5]. Post-contrast and pre-contrast images are then compared to identify CSF and contrast leak areas.

CT brain with positive contrast was performed in a 45-year-old male patient who presented with loss of consciousness to evaluate for SAH. CT brain with negative contrast was studied in a 40-year-old male patient with loss of consciousness after a road traffic accident to evaluate pneumocephalus. 

Routine 3D-CISS sequence MRI was studied for a patient with trigeminal neuralgia. 3D-CISS is a gradient echo MRI sequence that is useful in evaluating a variety of pathological states where regular MRI does not provide appropriate anatomical information. 3D-CISS sequences are highly useful in evaluating cranial nerves, CSF rhinorrhea, and cisternal spaces due to the detection of subtle CSF intensity lesions that may be missed on routine sequences. The superiority and higher sensitivity of the 3D-CISS sequence are attributable to the accentuation of T2 values between CSF and other structures [7,8].

## Review

Normal anatomy

The central nervous system (CNS) components are all encased within three layers known as the meninges. The innermost layer is known as the pia mater, which is closely adherent to the brain and follows the contours of the sulci and gyri on the cerebral cortex. The outermost layer is known as the dura mater, which lies in close contact with the periosteum. The middle layer contained between the dura and pia mater is known as the arachnoid mater (spiderweb-like), and it is loosely adherent to the brain [9-14]. The meninges define three distinct spaces. The epidural space exists between the skull and the dura mater. The subdural space is found between the dura and arachnoid mater. The subarachnoid space lies between the arachnoid and pia mater. The epidural and subdural spaces are potential spaces, whereas, the subarachnoid space contains CSF, major vascular structures, and the subarachnoid cisterns [9-13]. Due to the natural variation in the shape of the brain parenchyma and the loose adherence of the arachnoid mater to the brain, the subarachnoid space is nonuniform in depth within the CNS. Thus, the pia and arachnoid mater are not in close approximation to each other in certain places leading to the formation of naturally enlarged CSF-filled pockets known as the subarachnoid cisterns. The cisterns transmit various neurovascular structures, making them clinically and surgically significant. It is worth noting that although the subarachnoid cisterns are often described as distinct compartments, the cisterns are in free communication with each other and with the rest of the subarachnoid space. Thus, the cisterns are not actually anatomically separate [12,13]. The subarachnoid cisterns of the CNS are akin to the byzantine cisterns of ancient Rome built to store water [15,16].

Classification of cisterns

Cisterns can be anatomically classified based on their location as either supratentorial, at the level of the tentorium, or infratentorial and whether they are paired or unpaired cisterns [1,2,13,15,16]. Table 1 shows the classification of subarachnoid cisterns.

**Table 1:** Classification of the subarachnoid cisterns.

Supratentorial subarachnoid cisterns		Subarachnoid cisterns at the level of the tentorium		Infratentorial subarachnoid cisterns	
Paired	Unpaired	Paired	Unpaired	Paired	Unpaired
Olfactory cistern	Cisterna chiasmatica (chiasmatic cistern)	Cisterna ambiens (ambient cistern)	Cisterna interpeduncularis (interpeduncular cistern)	Cisterna pontocerebellaris (pontocerebellar cistern/prepontine cistern)	Cisterna magna
Carotid cistern					
Sylvian cistern	Cisterna laminae terminalis (cistern of lamina terminalis)		Cisterna quadrigeminalis (quadrigeminal cistern)	Medullary cistern	
Oculomotor cistern					
Crural cistern	Cisterna pericallosa (Pericallosal cistern)		Superior cerebellar cistern		

Schematic representation of the subarachnoid cisterns

**Figure 1:**
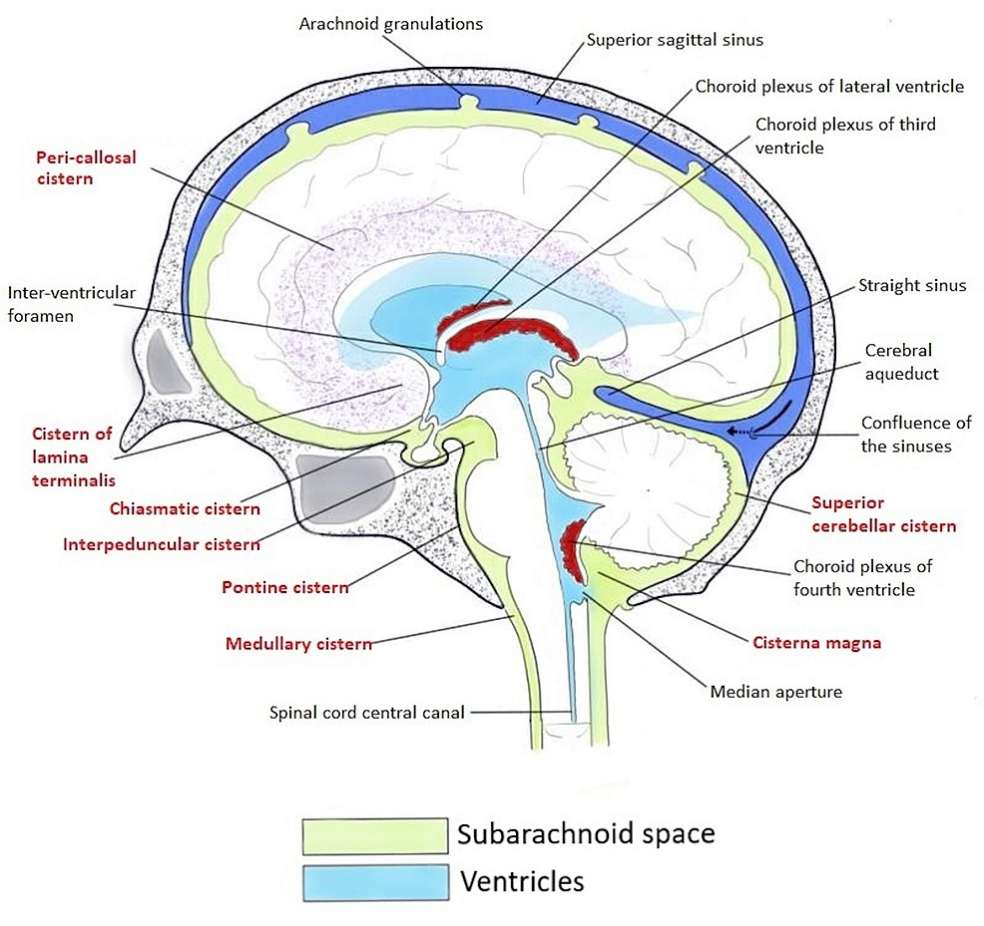
Schematic representation of the subarachnoid cisterns in the midsagittal plane.

**Figure 2:**
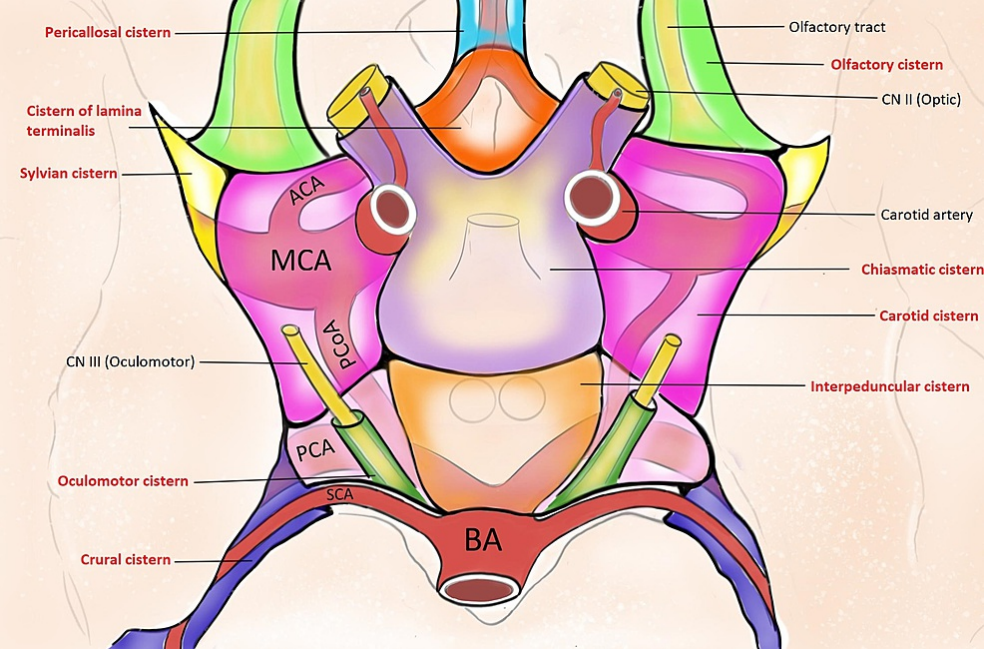
Schematic representation of the subarachnoid cisterns as seen from an inferior view. BA: Basilar Artery; SCA: Superior Cerebellar Artery; PCA: Posterior Cerebral Artery; PCoA: Posterior Communicating Artery; MCA: Middle Cerebral Artery; ACA: Anterior Cerebral Artery; CN: Cranial Nerve.

Figure 1 and Figure 2 show the schematic representations of the subarachnoid cisterns in the midsagittal plane (Figure 1) and from an inferior view (Figure 2).

Paired supratentorial cisterns

Olfactory Cisterns

**Figure 3:**
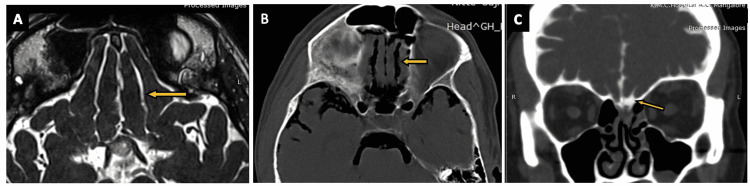
The olfactory cistern. (A) The olfactory cistern (arrow) as visualized on an axial section of the routine three-dimensional (3D) constructive interference in steady state (CISS) MRI sequence. (B) The olfactory cistern (arrow) as visualized on an axial section of CT brain (negative contrast) in a case of pneumocephalus. (C) The olfactory cistern (arrow) as visualized on a coronal section of CT cisternography in a case of suspected CSF rhinorrhea.

Olfactory cisterns (Figures 3A-3C) are triangular-shaped paired cisterns that can be seen in the coronal and axial sections (arrows in Figures 3A-3C), and are situated in the superficial portion of the olfactory sulcus. The olfactory cisterns are bordered laterally by the orbital gyrus and medially by the gyrus rectus [2,13,17]. The anterior portion of the cistern is located along the olfactory bulb, and the posterior portion of the cistern is located towards the clinoid process with medial and lateral extensions [17,18]. The anterior portion originates at the level of the anterior olfactory tentorium and the posterior portion extends to the olfactory trigone. The inferior portion of the cistern lies medially and superiorly to the internal carotid artery [18]. The vascular contents of the olfactory cistern include the fronto-orbital artery, orbital vein, and olfactory artery and vein. The neural contents of the olfactory cistern include the olfactory tract and bulb [2,13]. 

Carotid Cisterns

**Figure 4:**
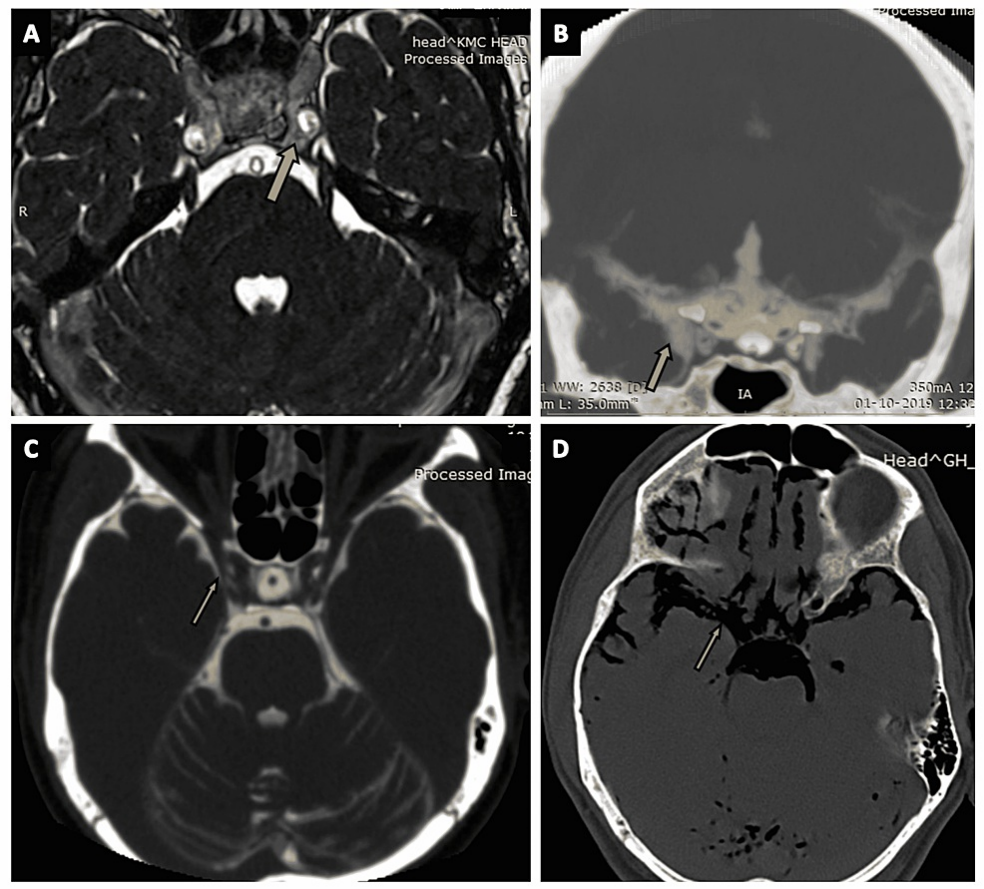
The carotid cistern. (A) The carotid cistern (arrow) located medial to the temporal lobe as seen on an axial section of the routine three-dimensional (3D) constructive interference in steady state (CISS) MRI sequence. (B) The carotid cistern (arrow) visualized on a coronal section of CT cisternography in a case of suspected CSF rhinorrhea. (C) The carotid cistern (arrow) as seen on an axial section of CT cisternography in a case of suspected CSF rhinorrhea. (D) The carotid cistern (arrow) as seen on an axial section of CT brain (negative contrast) in a case of pneumocephalus.

Carotid cisterns (Figures 4A-4D) are paired cisterns located anteriorly to the crural cisterns, between the ipsilateral optic nerve and internal carotid artery (ICA). Carotid cisterns lie medially to the temporal lobes and anterior clinoid process (arrows in Figures 4A-4D) The optic chiasm borders the carotid cistern anteriorly, medially, and posteriorly. The roof of the carotid cistern is formed by the anterior perforated substance, and the floor is formed by the dura of the cavernous sinus [19]. The contents of the carotid cisterns are the ICA, posterior communicating artery (PCoA), and ophthalmic artery. The origin of the anterior choroidal artery can also be found in the carotid cisterns [2,13]. 

Sylvian Cisterns

**Figure 5:**
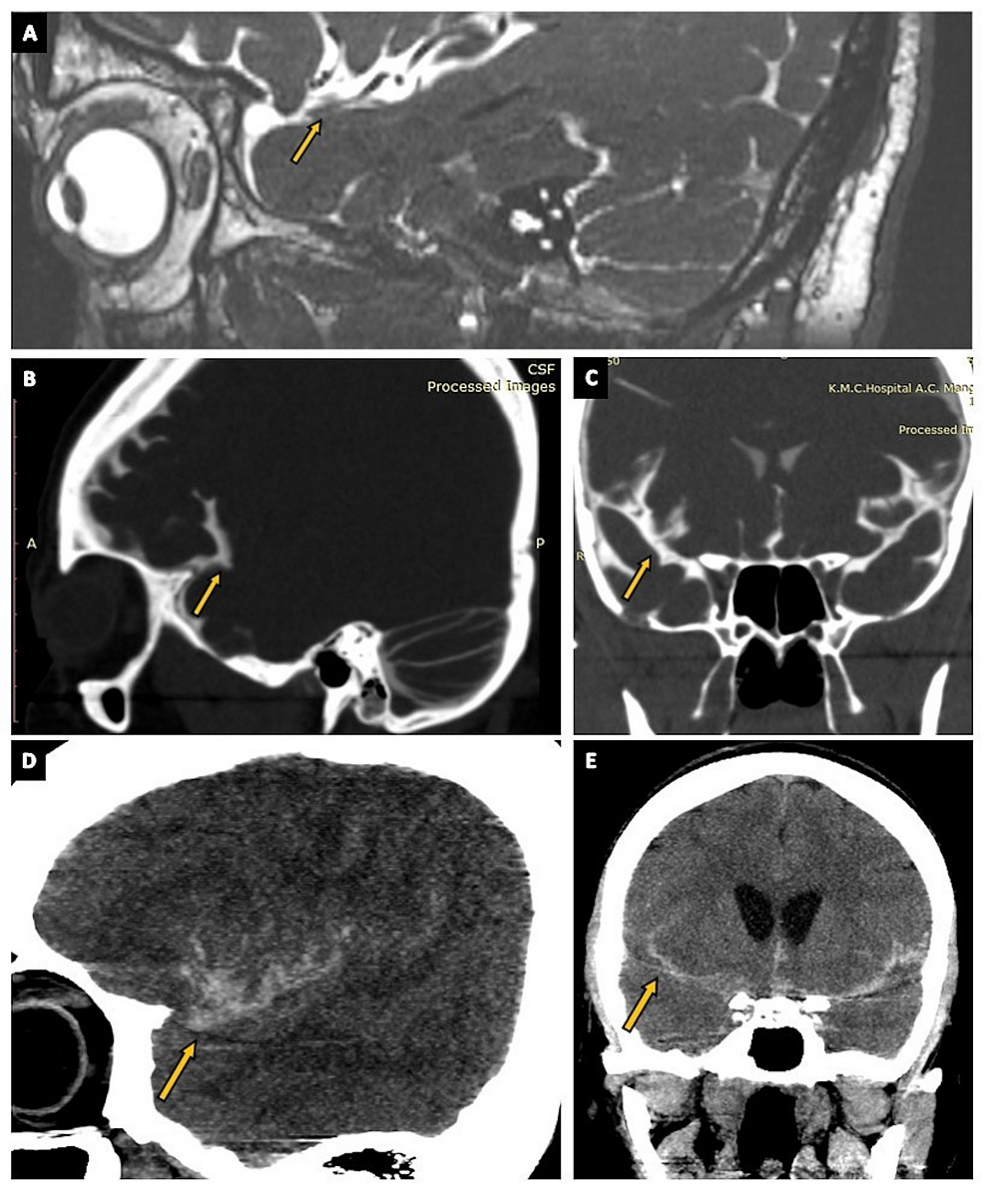
The Sylvian cistern. (A) The Sylvian cistern (arrow) as seen on a parasagittal section of the routine three-dimensional (3D) constructive interference in steady state (CISS) MRI sequence. (B) The Sylvian cistern (arrow) vizualised on a parasagittal section of CT cisternography in a case of suspected CSF rhinorrhea. (C) The Sylvian cistern (arrow) vizualised on a coronal section of CT cisternography in a case of suspected CSF rhinorrhea. (D) The Sylvian cistern (arrow) as seen on a parasagittal section of CT brain (positive contrast) in a case of subarachnoid hemorrhage (SAH). (E) The Sylvian cistern (arrow) as seen on a coronal section of CT brain (positive contrast) in a case of SAH.

**Figure 6:**
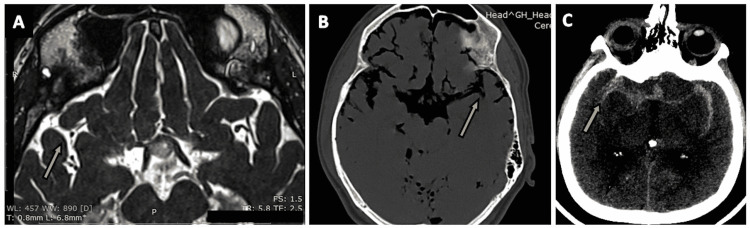
The Sylvian cistern (continued). (A) The Sylvian cistern (arrow) as seen on an axial section of the routine three-dimensional (3D) constructive interference in steady state (CISS) MRI sequence. (B) The Sylvian cistern (arrow) as seen on an axial section of CT brain (negative contrast) in a case of pneumocephalus. (C) The Sylvian cistern (arrow) as seen on an axial section of CT brain (positive contrast) in a case of subarachnoid hemorrhage (SAH).

Sylvian cisterns (Figures 5A-5E and Figures 6A-6C) are T-shaped paired CSF-filled pockets that serve as a transition space between the basal cisterns and the hemispheric subarachnoid space. The Sylvian cisterns are bounded by the insular and opercular cortex. These cisterns are compartmentalized into an anterior compartment, which extends laterally from the origins of the middle cerebral artery (MCA) to the limen insula, and a posterior compartment, which is located behind the limen insula [13,20]. Arterial contents are comprised of the MCA, origins of the lenticulostriate artery, anterior temporal artery, and temporopolar artery. Venous contents are comprised of the middle cerebral vein, superficial Sylvian, and deep Sylvian veins. Detailed knowledge of the Sylvian cistern’s anatomy is important to prevent damage to underlying neurovascular structures during microsurgical dissections of the Sylvian cistern in pterional operations [13,20-22].

Oculomotor Cisterns

**Figure 7:**
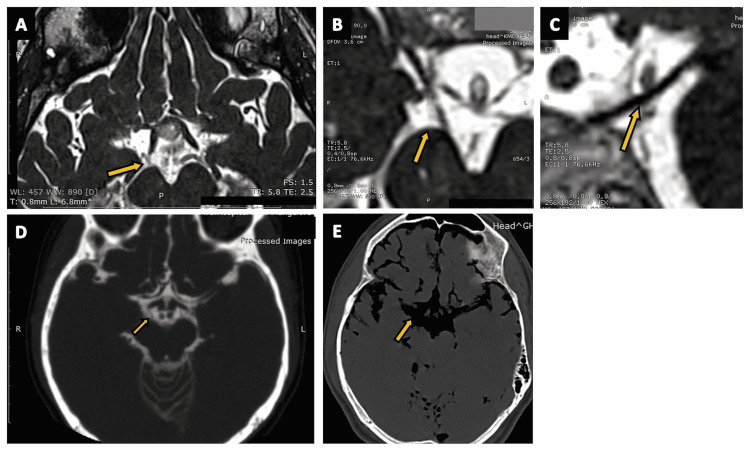
The oculomotor cistern. (A) The oculomotor cistern (arrow) as seen on an axial section of the routine three-dimensional (3D) constructive interference in steady state (CISS) MRI sequence. (B) A zoomed-in version of the oculomotor cistern (arrow) in an axial section of the routine 3D-CISS MRI sequence. (C) The oculomotor nerve (CN III) within the oculomotor cistern (arrow) is seen on an oblique section of the routine 3D-CISS MRI sequence. (D) The oculomotor cistern (arrow) visualized on an axial section of CT cisternography in a case of suspected CSF rhinorrhea. (E) The oculomotor cistern (arrow) as seen on an axial section of CT brain (negative contrast) in a case of pneumocephalus.

The oculomotor cisterns (Figures 7A-7E) are paired, small CSF-filled dural cuffs that surround the oculomotor nerve (CN III) as it enters the cavernous sinus (arrow in Figure 7C). It is worth noting that although the CN III enters the orbital apex after penetrating the cavernous sinus, the oculomotor cistern tapers and terminates at the anterior clinoid process [23-25]. The oculomotor cistern is a surgically relevant space as it is avascular and can be used to expose and manipulate CN III during cavernous sinus surgery [26].

Crural Cisterns

Crural cisterns are paired cisterns that are located between the cerebral peduncles medially and the uncus laterally [2,13,27]. The crural cisterns communicate with the Sylvian cistern dorsally [2]. The arterial contents of the crural cisterns are the anterior choroidal and medial posterior choroidal arteries. The basal vein of Rosenthal can also be found in the crural cisterns [2,13].

Unpaired supratentorial cisterns

Cisterna Chiasmatica

**Figure 8:**
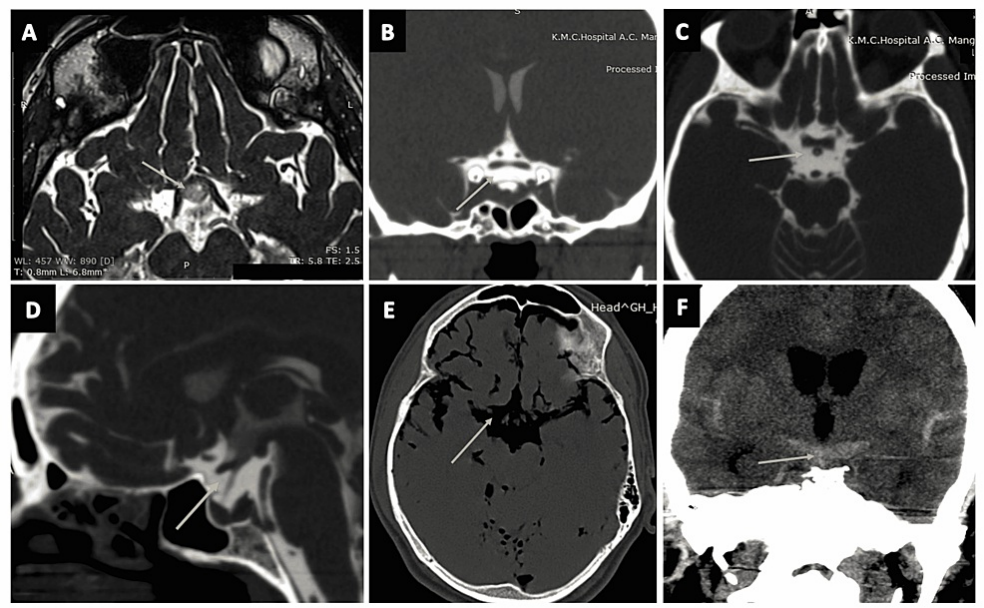
The cisterna chiasmatica (chiasmatic cistern). (A) The chiasmatic cistern (arrow) as seen on an axial section of the routine three-dimensional (3D) constructive interference in steady state (CISS) MRI sequence. (B), (C), and (D) show the chiasmatic cistern (arrows) as seen on the coronal, axial, and sagittal sections of CT cisternography, respectively, in a case of suspected CSF rhinorrhoea. (E) The chiasmatic cistern (arrow) visualized in the axial section of CT brain (negative contrast) in a case of pneumocephalus. (F) The chiasmatic cistern (arrow) as visualized on the coronal section of CT brain (positive contrast) in a case of subarachnoid hemorrhage (SAH).

The cisterna chiasmatica (chiasmatic cistern) is a midline structure (Figures 8A-8F) that is also known as the suprasellar cistern (situated superiorly to sella turcica) (arrow in Figure 8D). It lies between the uncus of the temporal lobes and under the hypothalamus. It communicates superiorly with the cistern of lamina terminalis, anterolaterally with the Sylvian cistern, and posteriorly with the interpeduncular cistern [2]. The chiasmatic cistern is separated from the interpeduncular cistern by the Liliequist membrane [28]. The neural contents of the chiasmatic cistern are the hypophyseal stalk, optic nerve (CN II), and optic chiasm. The arterial contents of the chiasmatic cistern are comprised of the perforating carotid artery branches, superior hypophyseal artery, and infundibular artery. The optic venous plexus can also be found within the chiasmatic cistern [2,9].

Cisterna Laminae Terminalis

**Figure 9:**
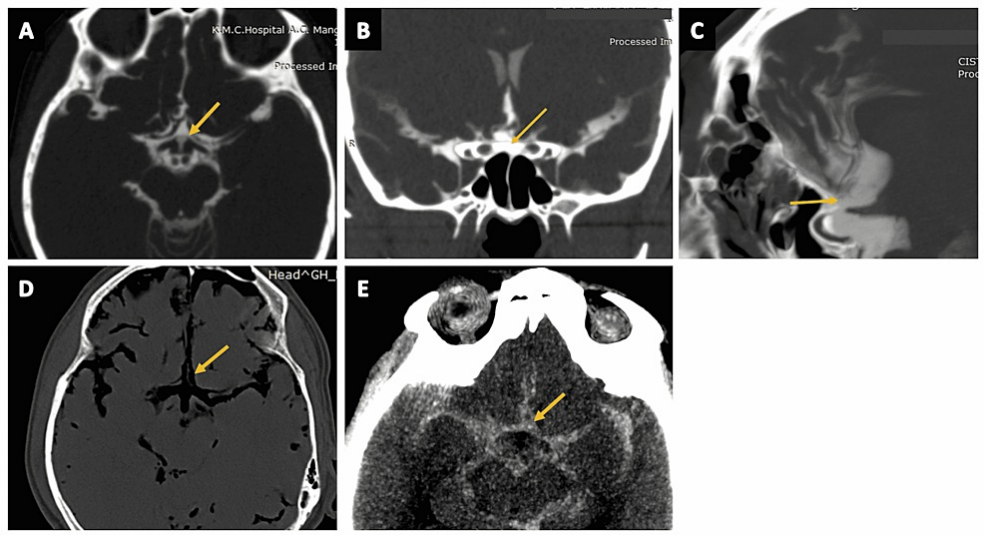
The cisterna laminae terminalis (cistern of lamina terminalis). (A), (B), and (C) show the cistern of lamina terminalis (arrows) as seen on the axial, coronal, and sagittal sections of CT cisternography, respectively, in a case of suspected CSF rhinorrhea. (D) The cistern of lamina terminalis (arrow) as seen on the axial section of CT brain (negative contrast) in a case of pneumocephalus. (E) The cistern of lamina terminalis (arrow) as visualized on the axial plane of CT brain (positive contrast) in a case of subarachnoid hemorrhage (SAH).

The cisterna laminae terminalis (cistern of lamina terminalis) (Figures 9A-9E) is a tent-shaped cistern (arrows in Figures 9A, 9D, 9E) is situated in the midline of the basal telencephalon (arrows in Figures 9A, 9B, 9D, 9E) [2,13,29]. The cistern of the lamina terminalis is bordered laterally by the medial surface of the posterior gyrus rectus and the septal area. Anteriorly, this cistern is bordered by the pia and arachnoid mater in front of the anterior communicating arteries. The optic chiasm forms this cistern’s inferior wall, and the lamina terminalis forms its posterior wall [2,13,29]. It forms a connection between the chiasmatic and pericallosal cistern. The contents of the cistern of lamina terminalis include the A1 and proximal A2 segments of the anterior cerebral artery (ACA), anterior communicating artery (ACoA), recurrent artery of Heubner, arteries of the hypothalamus, orbitofrontal arteries origin, and veins of the lamina terminalis [13,29,30]. Since this cistern contains most of the components of the anterior circulation system, it is also clinically and surgically important as it is a common site of aneurysm formation [2].

Cisterna Pericallosa

**Figure 10:**
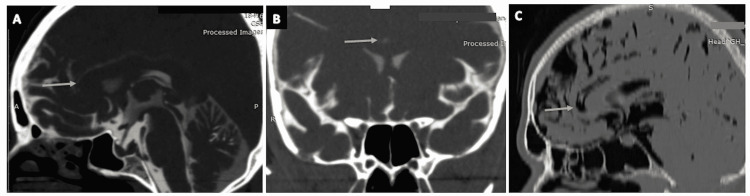
The cisterna pericallosa (pericallosal cistern). (A) and (B) show the pericallosal cistern (arrows) as seen on the sagittal and coronal sections of CT cisternography, respectively in a case of suspected CSF rhinorrhoea. (C) The pericallosal cistern (arrow) as seen on the sagittal section of CT brain (negative contrast) in a case of pneumocephalus.

When viewed in the sagittal plane, the cisterna pericallosa (pericallosal cistern) has a convex shape to it (arrows in Figures 10A, 10C); extending under the falx cerebri between the cerebral hemispheres and above the corpus callosum (arrows in Figures 10A-10C) [2]. It lies superior to the cistern of lamina terminalis and corpus callosum, connects with the chiasmatic cistern, and encases the peri-callosal artery. The contents of the callosal cistern can be divided into contents in the anterior and posterior portions of the cistern. In the anterior portion of the pericallosal cistern, the contents are - ACA (A2 segment), MCA, proximal medial striate artery, recurrent artery of Heubner, origins of the frontopolar, fronto-orbital, and callosomarginal arteries. The anterior portion of the pericallosal cistern also contains the anterior cerebral and orbital veins. The posterior portion of the pericallosal cistern contains the posterior pericallosal artery and vein, and occipital veins [9].

Paired cisterns at the level of the tentorium

Cisterna Ambiens

**Figure 11:**
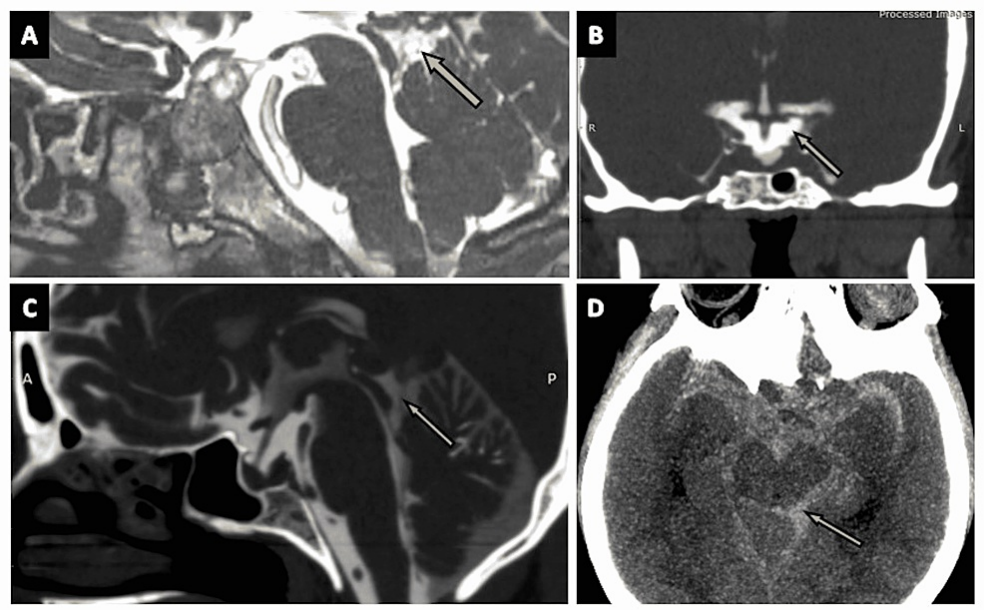
The cisterna ambiens (ambient cistern). (A) The ambient cistern (arrow) as seen on the sagittal section of the routine three-dimensional (3D) constructive interference in steady state (CISS) MRI sequence. (B) and (C) show the ambient cistern (arrows) in the coronal and sagittal planes of CT cisternography, respectively, in a case CSF rhinorrhoea. (D) The ambient cistern (arrow) as seen on the axial section of CT brain (positive contrast) in a case of subarachnoid hemorrhage (SAH).

The cisterna ambiens (ambient cistern) (Figures 11A-11D) is surgically significant as it is situated along the lateral aspect of the brain stem (arrows in Figures 11B, 11D) with both supratentorial and infratentorial extensions. The superior cerebellar membrane divides the ambient cistern into a superior compartment (posterior cerebellar ambient cistern) and an inferior compartment (superior cerebellar ambient cistern) [31,32]. The superior compartment (posterior cerebellar ambient cistern) carries the posterior cerebral artery (PCA), medial and lateral posterior choroidal arteries, perforating branches to the brain stem and the basal vein of Rosenthal. The inferior compartment (superior cerebellar ambient cistern) carries the superior cerebellar artery (SCA) and the trochlear nerve (CN IV) [2,3,13].

Unpaired cisterns at the level of the tentorium

Cisterna Interpeduncularis

**Figure 12:**
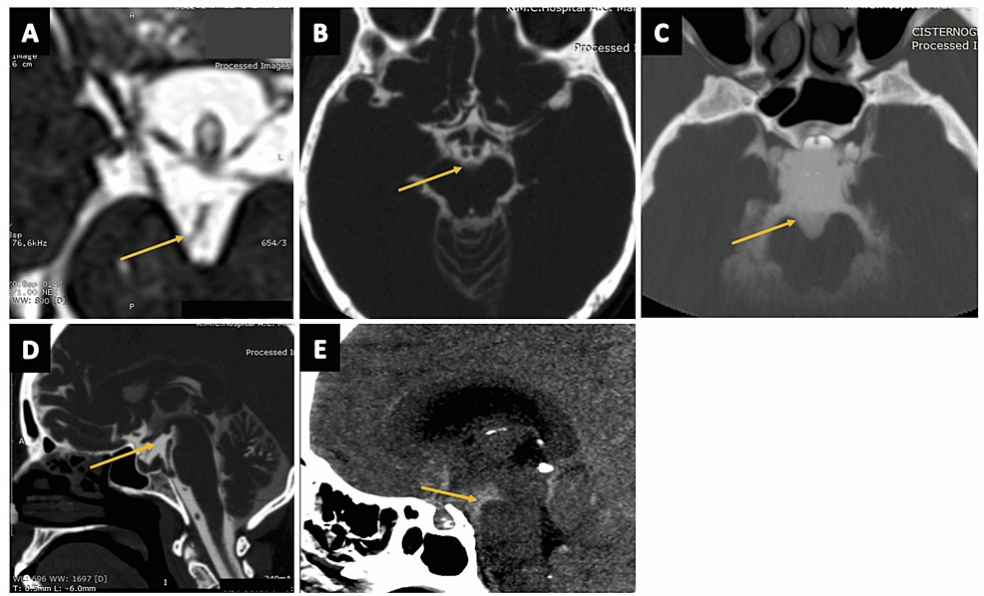
The cisterna interpeduncularis (interpeduncular cistern). (A) A zoomed-in portion of an axial section of the routine three-dimensional (3D) constructive interference in steady state (CISS) MRI sequence highlighting the interpeduncular cistern (arrow). (B), (C), and (D) show the interpeduncular cistern (arrows) in the axial, oblique-axial, and sagittal sections of CT cisternography, respectively, in a case of suspected CSF rhinorrhoea. (E) The interpeduncular cistern (arrow) as seen on the sagittal plane of CT brain (positive contrast) in a case of subarachnoid hemorrhage (SAH).

**Figure 13:**
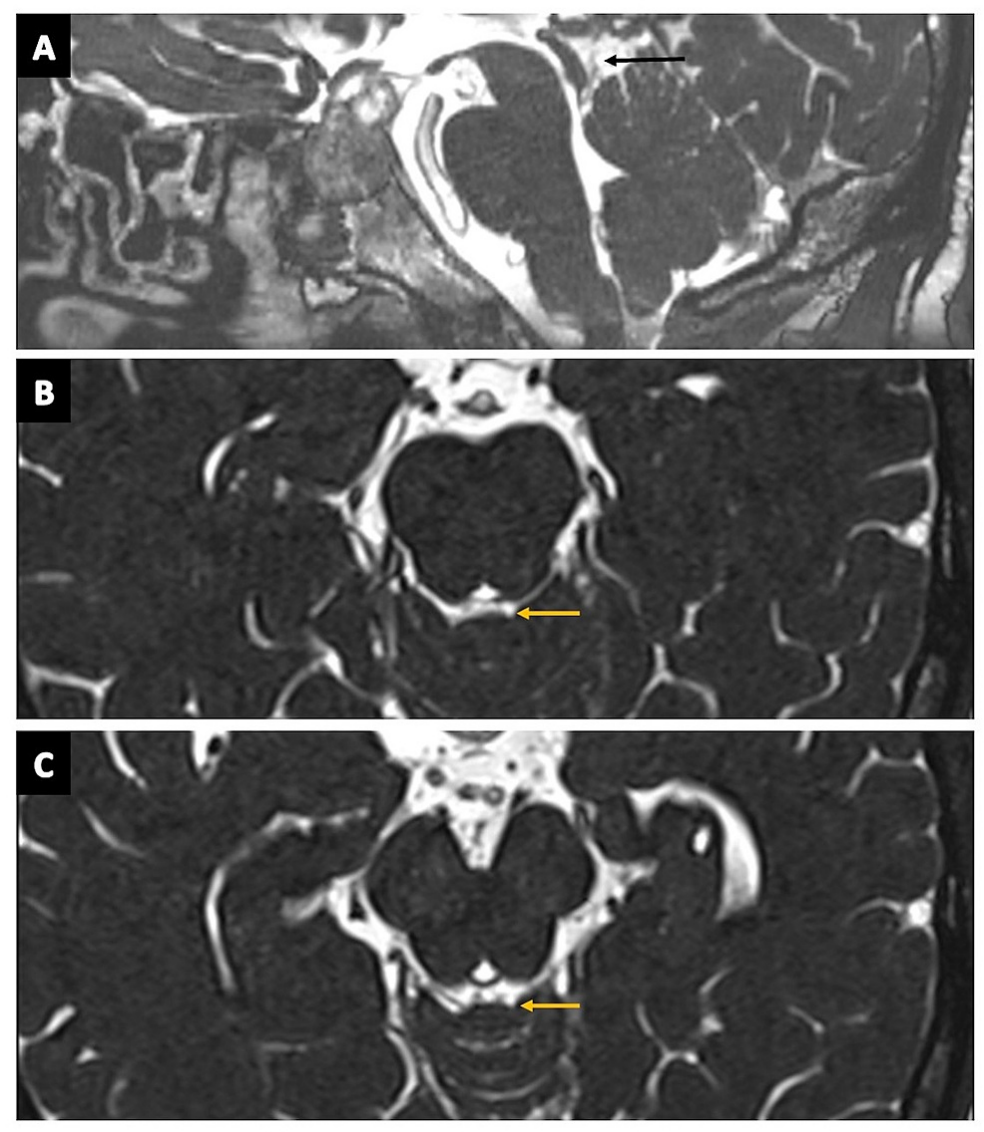
The cisterna quadrigeminalis (quadrigeminal cistern). (A) The quadrigeminal cistern (arrow) in the sagittal section of the three-dimensional (3D) constructive interference in steady state (CISS) MRI sequence. (B) and (C) show the quadrigeminal cistern (arrows) as seen on the axial planes of 3D-CISS MRI sequence.

Cisterna interpeduncularis (interpeduncular cistern), also known as the intercrural cistern, (Figures 12A-13E) is a conically shaped cistern found in the interpeduncular fossa (arrows in Figures 12A-12C). It is made by the convergence of the subarachnoid space of the supratentorial and infratentorial regions and is limited anteriorly by the sella turcica, pituitary stalk, and the optic chiasm (arrows in Figures 12D, 12E). The roof of this cistern is formed by the inferior surface of the mesencephalon, the lower diencephalon, and the mammillary bodies. Medial and lateral pontomesencephalic membranes border the interpeduncular cistern inferiorly [33]. The division of basilar artery, peduncular segments of the PCA and SCA, thalamogeniculate arteries, and medial and lateral posterior choroidal arteries are present in it. The basal vein of Rosenthal and the oculomotor nerve (CN III) can also be found in the interpeduncular cistern [2,13].

Cisterna Quadreigeminalis

**Figure 14:**
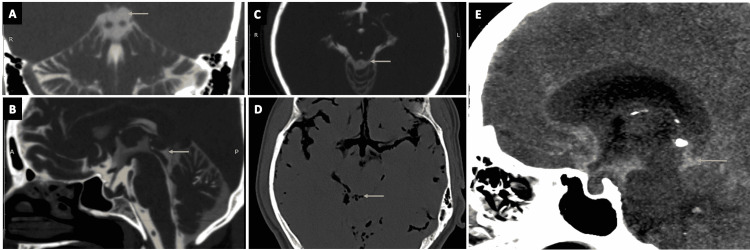
The cisterna quadrigeminalis (quadrigeminal cistern) (continued). (A), (B), and (C) show the quadrigeminal cistern (arrows) as visualized in the coronal, sagittal, and axial sections of CT cisternography, respectively, in a case of suspected CSF rhinorrhoea. (D) The quadrigeminal cistern (arrow) as seen in an axial plane of CT brain (negative contrast) in a case of pneumocephalus. (E) The quadrigeminal cistern (arrow) as seen on the sagittal plane of CT brain (positive contrast) in a case of subarachnoid hemorrhage (SAH).

Bordered anteriorly by the dorsal mesencephalon, the quadrigeminal plate, and the pineal gland, posteriorly by the vermis, superiorly by the splenium, and inferiorly by the collicular bodies and lingula of the cerebellum, the cisterna quadrigeminalis (quadrigeminal cistern) (Figures 13A-13C and Figures 14A-14E) is formed by medial extension of the cisterna ambiens [2]. This cistern comprises various vascular structures and the trochlear nerve (CN IV). The PCA and its perforating branches, posterior pericallosal arteries, the third part of the SCA, and the medial and lateral posterior choroidal arteries are present within the quadrigeminal cistern. Venous structures located in this cistern include the great cerebral vein of Galen, the basal vein of Rosenthal, internal cerebral veins, atrial veins, posterior pericallosal veins, and vein of the cerebellum mesencephalic fissure [2,13]. 

Superior Cerebellar Cistern

**Figure 15:**
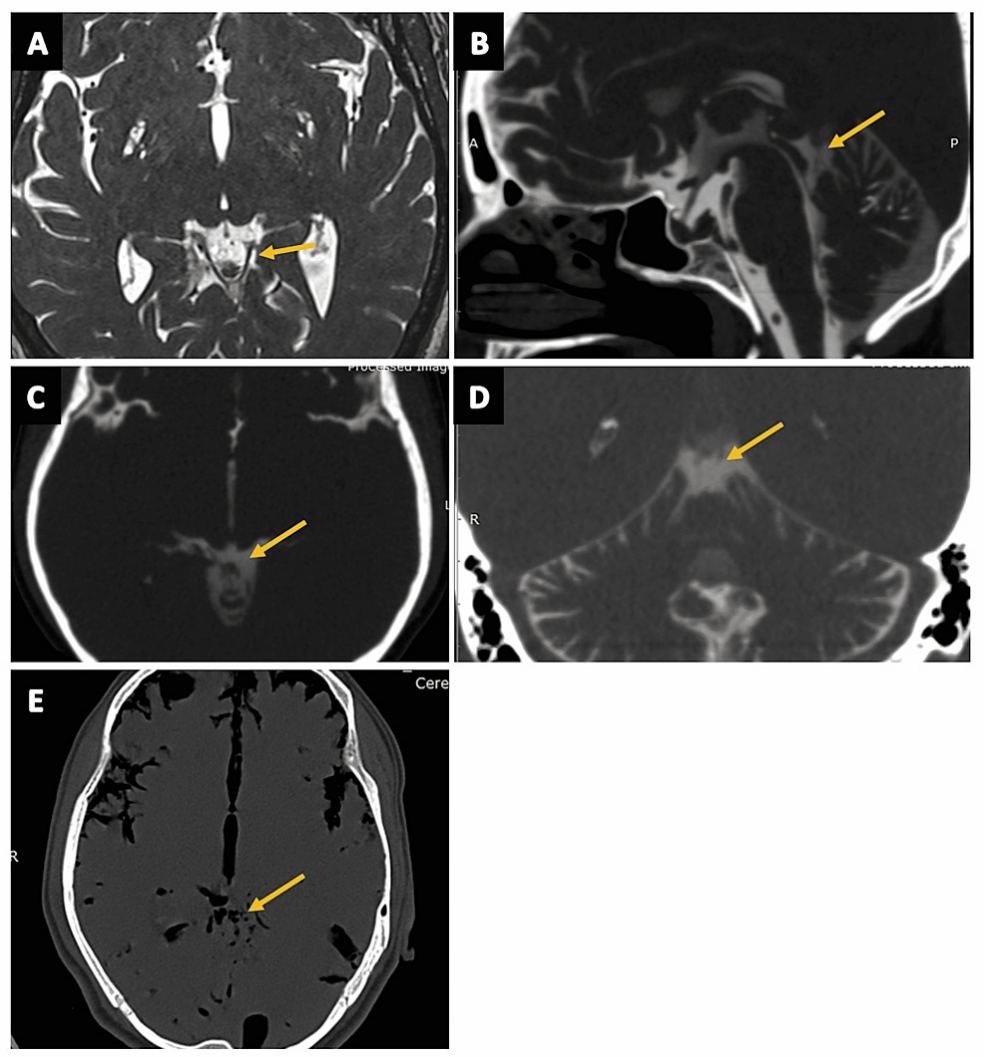
The superior cerebellar cistern. (A) The superior cerebellar cistern (arrow) as seen on the axial section of the three-dimensional (3D) constructive interference in steady state (CISS) MRI sequence. (B), (C), and (D) show the superior cerebellar cistern (arrows) as seen on the sagittal, axial, and coronal planes of CT cisternography, respectively, in a case of suspected CSF rhinorrhea. (E) The superior cerebellar cistern (arrow) as visualized in an axial plane of CT brain (negative contrast) in a case of pneumocephalus.

The superior cerebellar cistern (Figures 15A-15E) is located at the level of superior surface of the vermis, posteriorly to the ambient cistern (arrows in Figures 15B, 15D). It contains the SCA, superior cerebellar vein, and superior vermian veins [2,11,13].

Paired infratentorial cisterns

Cisterna Pontocerebellaris

**Figure 16:**
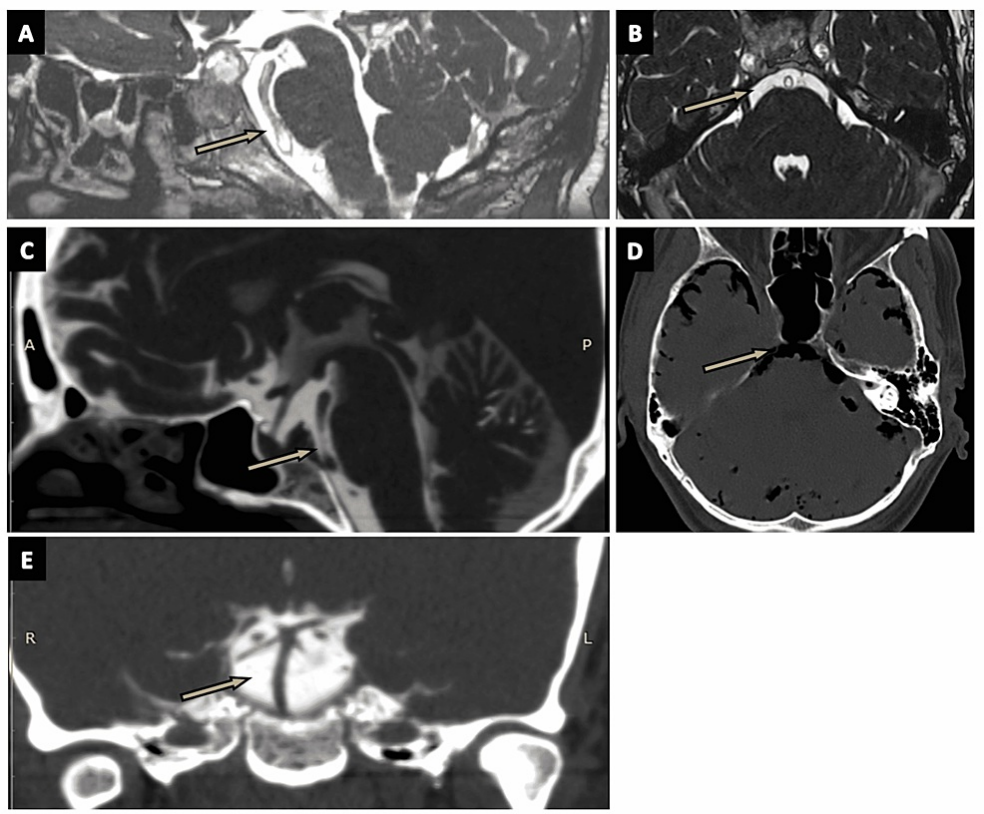
The cisterna pontocerebellaris (prepontine cistern, pontocerebellar cistern). (A) and (B) show the cisterna pontocerebellaris cistern (arrows) in the sagittal and axial sections of the routine three-dimensional (3D) constructive interference in steady state (CISS) MRI sequence, respectively. (C) The cisterna pontocerebellaris (arrow) in the sagittal plane of CT cisternography in a case of suspected CSF rhinorrhea. (D) The cisterna pontocerebellaris (arrow) in the axial section of CT brain (negative contrast) in a case of pneumocephalus. (E) The cisterna pontocerebellaris (arrow) in a coronal section of CT cisternography in a case of suspected CSF rhinorrhea.

The cisterna pontocerebellaris (also known as the prepontine cistern or the pontocerebellar cistern) is a rhomboid-shaped space encasing the pons (Figures 16A-16E), located at the pontocerebellar angle, and serves as an important surgical access landmark to infratentorial pathologies. The prepontine cistern has communications with the interpeduncular cistern (superiorly), the medullary cistern (inferiorly), and the chiasmatic cistern (anteriorly) [2,13]. The anterior inferior cerebellar artery (AICA) membrane separates prepontine and medullary cisterns. The superior cerebellar membrane limits the prepontine cistern superiorly and separates it from the ambient cistern [2,34]. This cistern has numerous neural and vascular contents including - CN IV (trochlear nerve), CN V (trigeminal nerve), CN VI (abducens nerve), CN VII (facial nerve), CN VIII (vestibulocochlear nerve), CN IX (glossopharyngeal nerve), CN X (vagus nerve), CN XI (accessory nerve), CN XII (hypoglossal nerve) basilar artery and plexus, AICA, superior cerebellar artery, posterior-inferior cerebellar artery (PICA), and the superior petrosal vein [2,13]. 

Medullary Cistern

**Figure 17:**
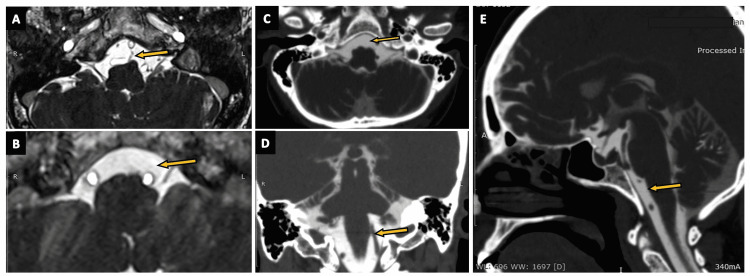
The medullary cistern. (A) The medullary cistern (arrow) in the axial plane of the three-dimensional (3D) constructive interference in steady state (CISS) MRI sequence. (B) A zoomed-in portion of an axial plane of the 3D-CISS MRI sequence showing the medullary cistern (arrow). (C), (D), and (E) show the medullary cistern (arrows) in the axial, coronal, and sagittal sections of CT cisternography, respectively, in a case of suspected CSF rhinorrhea.

The medullary cistern (Figures 17A-17E) encases the medulla oblongata (arrows in Figures 17A-17E). It communicates with the cisterna magna via the cerebello-medullary fissure [2,27]. It is separated from the pontocerebellar cistern by the AICA membrane. This cistern contains the glossopharyngeal nerve (CN IX), vagus nerve (CN X), and accessory nerve (CN XI). It also contains the PICA, lateral medullary vein, pontomedullary vein, and transverse medullary vein [2].

Unpaired infratentorial cisterns

Cisterna Magna

**Figure 18:**
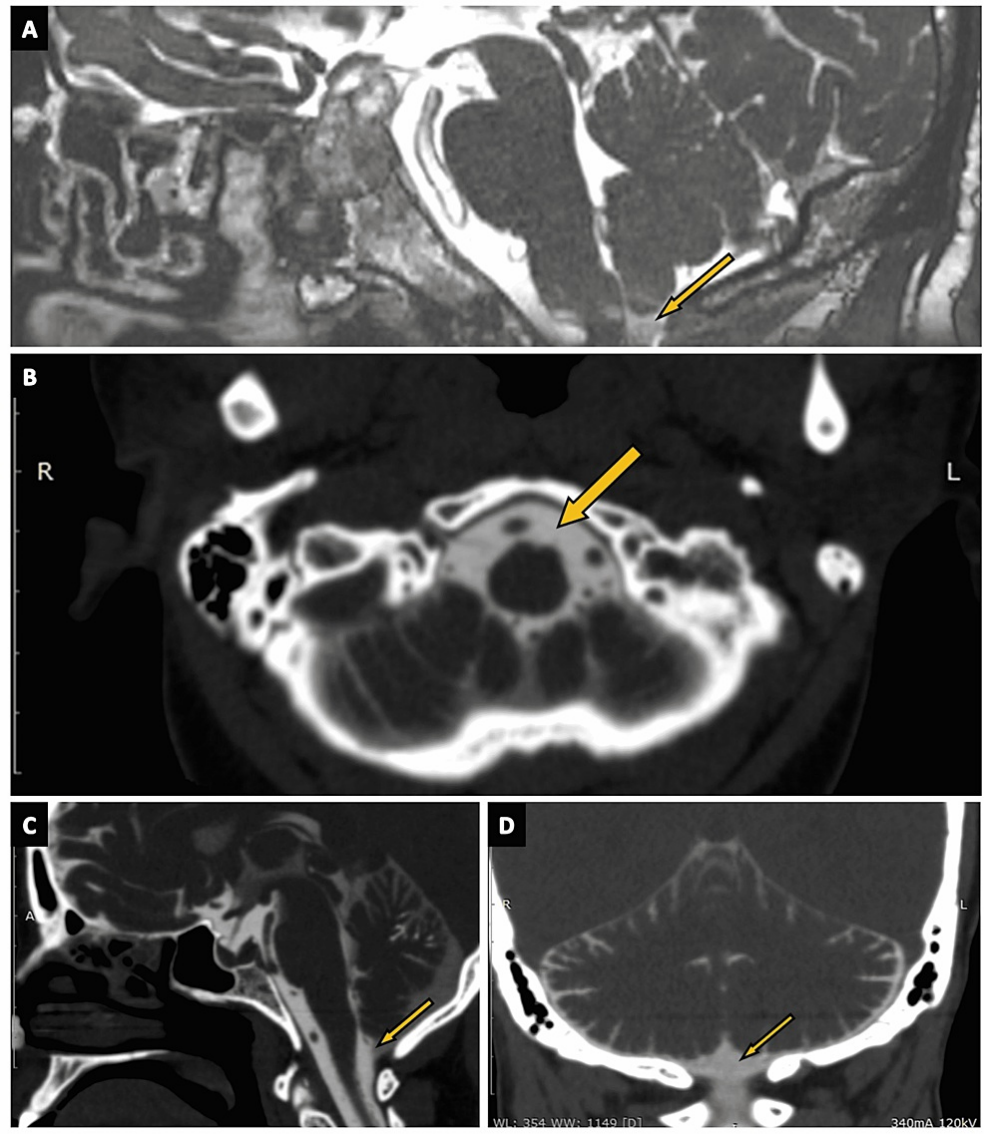
The cisterna magna. (A) The cisterna magna (arrow) in the sagittal plane of the three-dimensional (3D) constructive interference in steady state (CISS) MRI sequence. (B), (C), and (D) show the cisterna magna (arrows) as seen in the axial, sagittal, and coronal planes of CT cisternography, respectively, in a case of CSF rhinorrhea.

Cisterna magna (Figures 18A-18D) is the biggest of all subarachnoid cisterns and is located in the midline under the cerebellum and behind the medulla (arrows in Figures 18A-18D) [2,27]. It has a superior communication with the superior cerebellar cistern, and an inferior communication with the fourth ventricle (arrow in Fig. 18C) (via the foramen of Magendie and Luschka) [2]. The PICA membrane divides cisterna magna into the vallecular cistern (medial compartment) and PICA cistern (lateral compartment). The vallecular cistern houses the fifth segment of the PICA, whereas the third and fourth segments of PICA lie in the PICA cistern [2,13]. Other vascular structures inside the cisterna magna include the vertebral arteries, inferior vermian vein, medial posterior medullary vein, and the vein of the cerebello-medullary fissure. The cisterna magna also contains the glossopharyngeal nerve (CN IX), vagus nerve (CN X), and the accessory nerve (CN XI) [2,13].

## Conclusions

We believe that this study provides the necessary and additional information about the subarachnoid cisterns, which can be relevant to the radiologists, neurologists, and neurosurgeons to better understand, diagnose, and manage various neurological disorders. The detailed knowledge about the subarachnoid cisterns is also highly relevant in undergraduate and postgraduate medical teaching. The cisterns are akin to the byzantine cisterns of ancient Rome but have been simplified in this article using special radiological imaging circumstances.
